# Incidence and associated morbidity of sarcopenia in non-malignant small and large bowel anastomosis: propensity score-matched analysis

**DOI:** 10.1007/s00384-023-04441-6

**Published:** 2023-06-02

**Authors:** Luke Traeger, Sergei Bedrikovetski, Thuy-My Nguyen, James W. Moore, Tarik Sammour

**Affiliations:** 1 Colorectal Unit, Department of Surgery, Royal Adelaide Hospital, Adelaide, SA 5000 Australia; 2 Adelaide Medical School, Faculty of Health and Medical Sciences, University of Adelaide, Adelaide, SA 5000 Australia

**Keywords:** Sarcopenia, Non-malignant, Colorectal, Surgery

## Abstract

**Purpose:**

Sarcopenia is a prognostic factor for poor outcomes in colorectal cancer, but data are scarce in colorectal surgery for benign conditions where patients could benefit from a deferral of surgery to enter a prehabilitation programme. We assessed the incidence of sarcopenia and complications in patients with benign colorectal disease.

**Methods:**

Patients who underwent elective non-malignant colorectal surgery during 2018–2022 were retrospectively identified. The cross-sectional psoas area was calculated using computed tomography (CT) imaging mid-3^rd^ lumbar vertebrae. Sarcopenia was determined using gender-specific cut-offs. The primary outcome was complications measured by the comprehensive complication index (CCI).

**Results:**

Of 188 patients identified, 39 (20.7%) were sarcopenic. Patients diagnosed with sarcopenia were older (63 vs. 58 years, *p* = 0.047) and had a reduced BMI (24.7 vs. 27.38 kg/m^2^, *p* = 0.001). Sarcopenic patients had more complications (82.1 vs. 64.4%, *p* = 0.036), and CCI was statistically but not clinically higher (20.9 vs. 20.9, *p* = 0.047). On univariate linear regression analysis, age ≥ 65 years old, ASA grade ≥ 3, active smokers, sarcopenia, and preoperative anaemia were predictive of CCI. Propensity score-matched analysis was performed, matching 78 cases to remove selection bias, which demonstrated sarcopenia had no impact on postoperative complications. On multivariate analysis, age (*p* = 0.022), smoking (*p* = 0.005), and preoperative anaemia (*p* = 0.008) remained predictive of CCI.

**Conclusion:**

Sarcopenia is prevalent in one-fifth of patients undergoing benign colorectal surgery. Taking advantage of the longer preoperative waiting periods, sarcopenia could be explored as a target for prehabilitation programmes to improve outcomes.

## Introduction

Sarcopenia is the progressive age-related loss of skeletal muscle mass, quality and loss of function [1, 2]. In our ageing population, sarcopenia is becoming an increasingly recognised predictor of poor outcomes following colorectal surgery for malignant pathology [3–6]. Despite increased awareness, a preventative treatment strategy remains unclear [7].

Not only is malignancy more common in the elderly, but it also accelerates the development of sarcopenia through multiple mechanisms: increased cancer catabolism, malnutrition, immobilisation and the complications of treatment with neoadjuvant therapy [1, 2]. In a recent meta-analysis, sarcopenia was prevalent in 37% of colorectal cancer patients, significantly prolonging the length of hospital stay and increasing postoperative complications and mortality rates [4]. The increase in complications places a significant financial burden on the healthcare systems [8, 9]. Additionally, sarcopenia has also been linked with poor long-term outcomes, such as variable response to chemoradiotherapy, affecting the overall survival of patient’s [1, 10, 11].

Sarcopenia, and its associated adverse outcomes, are not restricted to oncology patients alone. In inflammatory bowel disease, studies have shown that 46.2% of patients are sarcopenic, primarily due to nutritional imbalance secondary to inflammatory mediators, poor caloric intake and malabsorption [6]. Additional to inflammatory bowel disease, sarcopenia has been reported as a significant prognostic indicator for poorer outcomes in chronic liver disease and pancreatitis [6].

To improve the outcomes of sarcopenic patients, prehabilitation programmes have been proposed to modify low muscle mass through nutritional support and physical activity. However, to date, there are limited reports of interventions that have been successful in modifying sarcopenia as a preoperative risk factor [7]. We hypothesise that the main barrier to successful prehabilitation is patient cohort selection. Although they have significant sarcopenia rates, patients with colorectal malignancies and severe inflammatory bowel disease have many hurdles to productive participation. Due to the side effects of neoadjuvant treatment, strict adherence to physical therapy and nutritional programmes may be hindered. Also, delaying urgent surgery in cases of colorectal malignancy or severe inflammatory bowel disease may not always be possible. On the other hand, there is potential to delay surgery to undertake prehabilitation in benign colorectal cases such as the reversal of stomas or for diverticular disease, and in fact, many of these patients wait for long periods anyway due to waitlist pressures in the public sector.

Despite the detrimental sequelae of sarcopenia in colorectal cancer and severe inflammatory bowel disease, a paucity of data remains on sarcopenia in benign colorectal conditions. Thus, this study aimed to assess the role of sarcopenia as a prognostic indicator for morbidity following non-malignant colorectal surgery.

## Methods

This study is reported using the Strengthening The Reporting of Observational studies in Epidemiology (STROBE) guidelines [12]. A waiver of consent for retrospective studies was approved by the Central Adelaide Local Health Network (CALHN) Human Research Ethics Committee.

### Patient selection

Patients who underwent elective colorectal surgery for non-malignant conditions under the Colorectal Unit of the Royal Adelaide Hospital (RAH), South Australia, between January 2018 and September 2022 were identified through an audit of elective theatre booking lists. Consecutive patients over 18 years old who underwent large or small bowel surgery were included. Non-malignant indications for surgery were included, such as inflammatory bowel disease, diverticular disease or non-inflammatory conditions such as restoration of intestinal continuity, irresectable polyp or polyposis and slow transit disease. Emergency surgery, elective surgery with category one clinical urgency (within 30 days), and patients with a diagnosis of malignancy were excluded. Patients were excluded if they had insufficient data or had not undergone computed tomography (CT) accessible by using our local Picture Archiving and Communication System (PACS) or InteleViewer™ Australia.

### Sarcopenia calculation

**Fig. 1:**
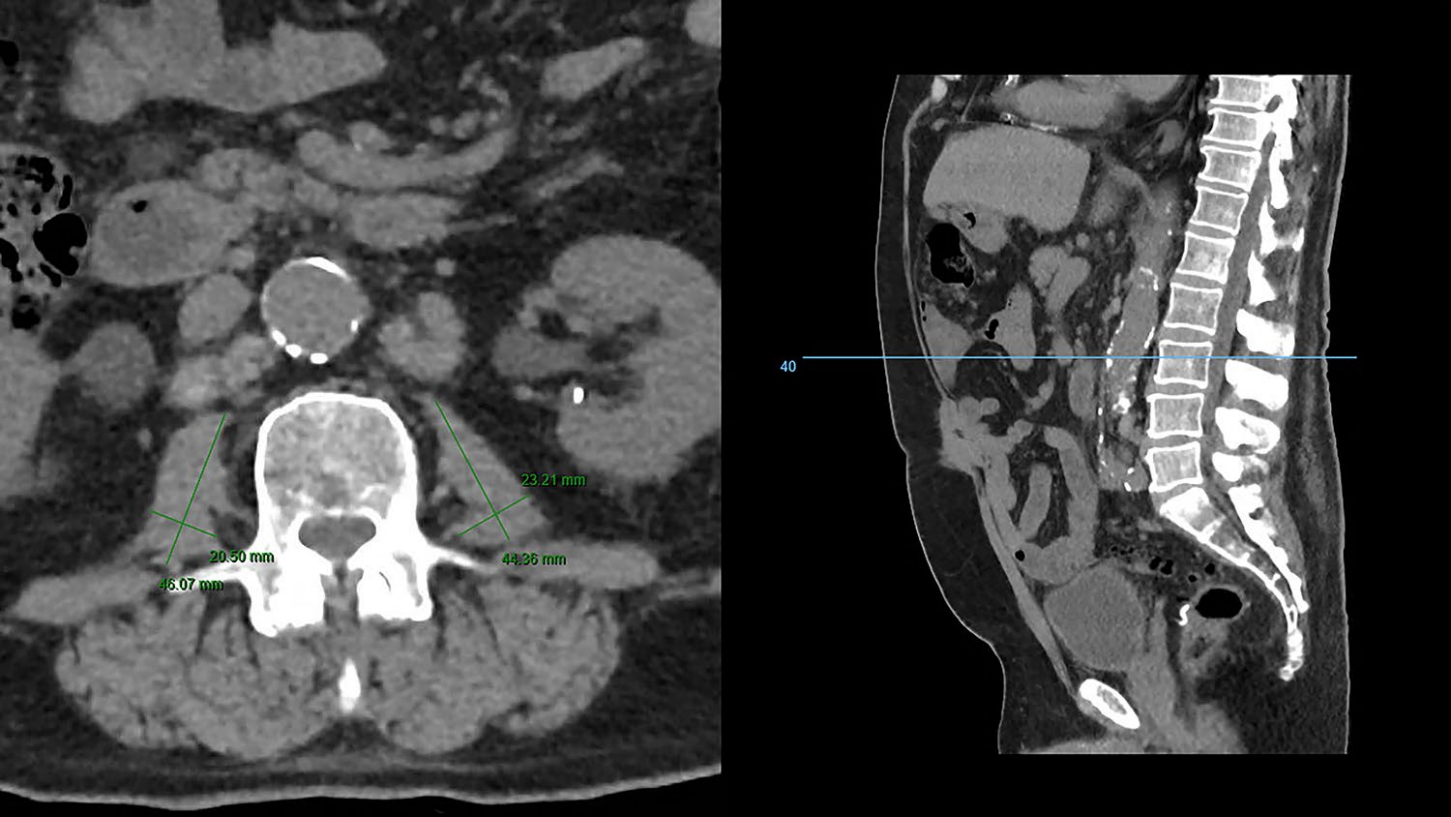
CT scans demonstrating total psoas area calculation at L3 vertebral level

Sarcopenia was defined using lean muscle mass as a surrogate marker. CT scans were retrieved and included if performed three months before surgery or in the immediate postoperative setting, whichever was later. Total psoas area (TPA) was calculated using the protocol defined by Jones et al. using CT scans multiplying the longest anterior–posterior and transverse muscle diameters bilaterally (Fig. 1) [13]. Calculation was performed mid-third lumbar vertebrae using PACS or InteleViewer™ Australia. TPA was indexed using the patient’s height squared (TPA mm^2^/m^2^) to calculate the total psoas area index (TPAI). Sarcopenia was defined using previously validated gender-specific cut-off points: < 385 mm^2^/m^2^ in females and < 545 mm^2^/m^2^ in males [14]. Two investigators (LT and SB) independently calculated TPA for all patients to identify sarcopenia, with conflicting diagnoses resolved by consensus.

### Data collection

Data were collected from electronic medical records retrospectively. Baseline demographics included age, gender, height, weight, body mass index (BMI) and American Society of Anaesthesiologists (ASA) score. Patient functional status and medical history of congestive cardiac failure, chronic obstructive pulmonary disease, diabetes mellitus and hypertension requiring medication were collated to form the modified frailty index (MFI)-5 [5]. Other nutritional data, including total protein and albumin, were also collected. Operative data were collected, including surgical approach (open/laparoscopic), conversion rates, procedure type, stoma formation and surgery duration.

### Outcomes

The primary outcome was the 30-day comprehensive complication index (CCI). This score measures overall morbidity, ranging from 0 (or no complication) to 100 (or death). The score was calculated by placing all the Clavien-Dindo (CD) grade complications that occurred in the 30-day postoperative period using the CCI online calculation (available at https://www.assessurgery.com/about_cci-calculator/, AssesSurgery GmbH, University of Zurich, Switzerland) [15, 16]. Secondary outcomes included highest CD grade, readmission and mortality rates and length of stay. Postoperative ileus was defined as not achieving GI2, a validated outcome measure comprised of time to first stool and tolerance of solid diet without significant nausea or vomiting by day four [17].

### Statistical methods

Statistical analysis was performed using SPSS 28.0 (SPSS Inc., Armonk, NY, USA). Shapiro–Wilk test was used to identify the parametricity of data with numerical data presented as median (IQR [range]) or mean (standard deviation). Univariate analysis was performed using the Mann–Whitney *U* for nonparametric variables or the Student *t* test for normally distributed continuous variables and the *χ*2 or Fisher’s exact test (when expected *n* < 5) for categorical variables. Propensity score-matched (PSM) analysis was performed to adjust for differences in preoperative characteristics. Variables of clinical relevance included age (continuous), BMI (categorical, ≥ 30 kg/m^2^), diabetes mellitus, pathology and resection side. Patients were matched 1:1 based on scoring, with a match tolerance of 0.25, with preference given to exact matches. Given the small sample size linear regression analyses were performed on all patients, after checking the data met all assumptions. Pre- and intraoperative variables were run through the univariate linear regression analysis to predict CCI. Statistically significant variables from the univariate linear regression analysis were then used for multivariate linear regression analyses. Statistical significance was met with a *P*-value < 0.05.

## Results

**Fig. 2:**
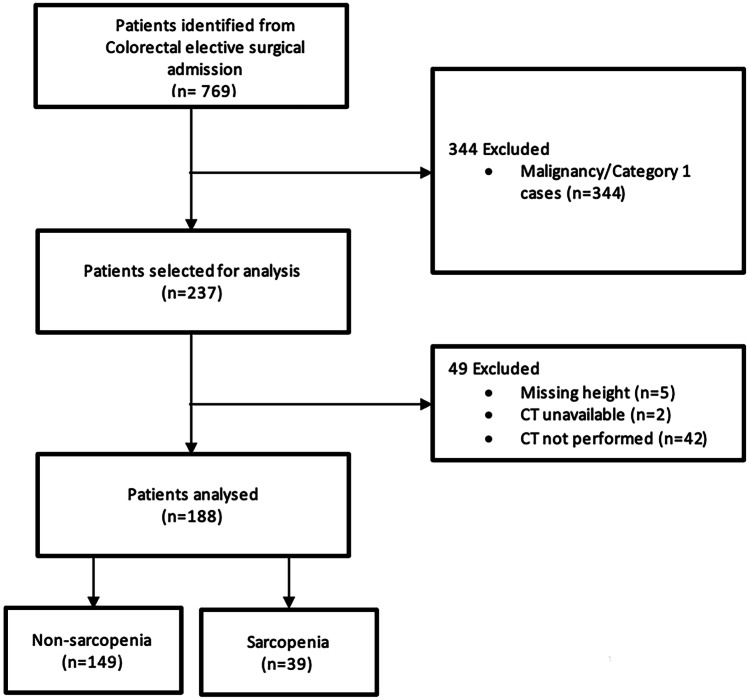
Patient selection flow chart

A total of 769 patients were retrospectively identified, of whom 188 were eligible for analysis (Fig. 2). Thirty-nine (20.7%) patients were classified as sarcopenic, and 149 were non-sarcopenic. The PSM cohort consisted of 34 exact matches and 5 fuzzy matches, totalling 78 patients.

Baseline characteristics are presented in Table 1. The sarcopenic group (SG) showed no predominance for gender; however, they had a lower BMI (24.71 vs. 27.38 kg/m^2^, *p* = 0.001) and weight when compared to the non-sarcopenic group (NSG) (73.96 vs. 83.04 kg, *p* < 0.001). TPAI was expectantly lower in the SG (378.46 vs. 743.03 mm^2^/m^2^, *p* < 0.001). There was no association with ASA grading, co-morbidities such as COPD, hypertension requiring medication or steroid use. The SG had a higher prevalence of diabetes mellitus (DM) requiring oral hypoglycaemic agents (25.6 vs. 8.7%, *p* = 0.021). After PSM, no statistical difference was demonstrated in baseline characteristics.

**Table 1:** Baseline characteristics comparing non-sarcopenic and sarcopenic patients. Values are median (range), mean (± standard deviation) or number (proportion)

	**Total** **(188)**	**Non-sarcopenic** **(*n* = 149)**	**Sarcopenic** **(*n* = 39)**	***p*-value**	**Matched non-sarcopenic** **(*n* = 39)**	**Matched sarcopenic** **(*n* = 39)**	***p*-value**
**Age; y**	59 (18–91)	58 (18–82)	63 (34–91)	**0.047**	61 (19–82)	63 (34–91)	0.472
**Gender**				0.356			0.241
Female	70 (37.2)	53 (35.6)	17 (43.6)		12 (30.8)	17 (43.6)	
Male	118 (62.8)	96 (64.4)	22 (56.4)		27 (69.2)	22 (56.4)	
**BMI; kg/m**^**2**^	26.81 (15.85–50.32)	27.38 (15.85–50.32	24.71 (17.44–33.80)	**0.001**	25.50 (17.24–36.21)	24.71 (17.44–33.80)	0.238
**Weight; kg**	81.16 (± 19.61)	83.04 (± 20.35)	73.96 (± 14.58)	**< 0.001**	75.12 (± 12.96)	73.96 (± 14.58)	0.356
**Body composition**							
TPA (mm^2^)	2062.02 (348.93–4667.25)	2160.44 (999.03–4667.25)	1104.41 (348.93–1913.69)	**< 0.001**	2084.69 (1099.79–4555.64)	1104.41 (348.93–1913.69)	**< 0.001**
TPAI (mm^2^/m^2^)	693.01 (120.74–1998.00)	743.03 (395.98–1998.00)	378.46 (120.74–544.10)	**< 0.001**	715.72 (476.02–1998.00)	378.46 (120.74–544.10)	**< 0.001**
**ASA**				0.609			0.648
I–II	108 (57.4)	87 (58.4)	21 (53.8)		23 (59.0)	21 (53.8)	
III–IV	80 (42.6)	62 (41.6)	18 (46.5)		16 (41.0)	18 (46.2)	
**Modified frailty index-5**				**0.010**			0.300
Low (0–1)	156 (83.0)	129 (86.6)	27 (69.2)		31 (79.5)	27 (69.2)	
High (> 2)	32 (17.0)	20 (13.4)	12 (30.8)		8 (20.5)	12 (30.8)	
**Smoking history**	43 (22.9)	31 (20.8)	12 (30.8)	0.187	7 (17.9)	12 (30.8)	0.187
**CCF**	2 (1.1)	0 (0.0)	2 (5.1)	**0.042**	0 (0.0)	2 (5.1)	0.152
**COPD**	19 (10.1)	13 (8.7)	6 (15.4)	0.219	3 (7.7)	6 (15.4)	0.481
**Hypertension**	59 (31.4)	45 (30.2)	14 (35.9)	0.495	20 (51.3)	14 (35.9)	0.171
**Diabetes mellitus**				**0.021**			0.250
Prescribed tablets	23 (12.2)	13 (8.7)	10 (25.6)		5 (12.8)	10 (25.6)	
Prescribed insulin	2 (1.1)	2 (1.3)	0 (0.0)		(0.0)	0 (0.0)	
**Prescribed regular steroids**	13 (6.9)	12 (8.1)	1 (2.6)	0.309	3 (7.7)	1 (2.6)	0.615
**Previous abdominal surgery**	144 (76.6)	112 (75.2)	32 (82.1)	0.405	28 (71.8)	32 (82.1)	0.282
**Preoperative haemoglobin; g/L**	136.69 (18.43)	137.60 (17.87)	133.21 (20.29)	0.093	134.64 (20.18)	133.21 (20.29)	0.380
**Preoperative total protein; g/L**	73 (49–91)	73 (56–86)	74 (49–91)	0.916	71 (57–83)	74 (49–91)	0.295
**Preoperative albumin; g/L**	37 (19–49)	37 (19–49)	38 (19–49)	0.815	35 (19–43)	38 (19–49)	0.281

*ASA* American Society of Anesthesiologists physical status, *BMI* body mass index, *CCF* congestive cardiac failure, *COPD* chronic obstructive pulmonary disease, *TPA* total psoas area, *TPAI* total psoas area index

Intraoperative characteristics were similar between the two groups, including the PSM cohort. As a whole cohort, the main indication for surgery was for non-inflammatory conditions (such as restoration of intestinal continuity and polyposis syndromes), followed by inflammatory bowel disease and diverticular disease (67.6 vs. 19.7 vs. 12.8%, respectively). Reversal of ileostomy (37.2%) and left-sided procedures (17.0%) accounted for the two most common surgical procedures. No differences were seen between the SG and NSG in the surgical approach, rates of conversion of laparoscopic to open, nor rates of stoma formation (Table 2).

**Table 2:** Intraoperative characteristics comparing non-sarcopenic and sarcopenic patients. Values are median (range) or number (proportion)

	**Total (*n* = 188)**	**Non-sarcopenic** **(*n* = 149)**	**Sarcopenic** **(*n* = 39)**	***P*-value**	**Matched non-sarcopenic** **(*n* = 39)**	**Matched sarcopenic** **(*n* = 39)**	***p*-value**
**Pathology/procedural type**				0.253			0.802
Diverticular	26 (13.8)	22 (14.8)	4 (10.3)		2 (5.1)	4 (10.3)	
IBD	37 (19.7)	33 (22.1)	4 (10.3)		5 (12.8)	4 (10.3)	
Non-inflammatory							
Restoration of intestinal continuity	105 (55.9)	80 (53.7)	25 (64.1)		25 (64.1)	25 (64.1)	
Polyposis/irresectable polyp	10 (5.3)	6 (4.0)	4 (10.3)		2 (5.1)	4 (10.3)	
Intolerable LARS, diarrhoea	6 (3.2)	5 (3.4)	1 (2.6)		2 (5.1)	1 (2.6)	
Slow transit/chronic constipation	2 (1.1)	2 (1.3)	0 (0.0)		2 (5.1)	0 (0.0)	
Other	2 (1.1)	1 (0.7)	1 (2.6)		1 (2.6)	1 (2.6)	
**Operations**				0.504			0.815
Formation of stoma (without resection)	5 (2.7)	3 (2.0)	2 (5.1)		1 (2.6)	2 (5.1)	
Hartmanns procedure	1 (0.5)	1 (0.7)	0 (0.0)		0 (0.0)	0 (0.0)	
Left-sided	32 (17.0)	27 (18.1)	5 (12.8)		7 (17.9)	5 (12.8)	
Right-sided	29 (15.4)	24 (16.1)	5 (12.8)		6 (15.4)	5 (12.8)	
Reversal of Hartmann’s	29 (15.4)	21 (14.1)	8 (20.5)		5 (12.8)	8 (20.5)	
Reversal of ileostomy	70 (37.2)	53 (35.6)	17 (43.6)		17 (43.6)	17 (43.6)	
SB procedure	5 (2.7)	4 (2.7)	1 (2.6)		0 (0.0)	1 (2.6)	
Total	17 (9.0)	16 (10.7)	1 (2.6)		3 (7.7)	1 (2.6)	
**Surgical approach**				0.574			> 0.999
Open	143 (76.1)	112 (75.2)	31 (79.5)		30 (76.9)	31 (79.5)	
Laparoscopic	45 (23.9)	37 (24.8)	8 (20.5)		9 (23.1)	8 (20.5)	
**Conversion from laparoscopic to open**	7 (15.6)	6 (16.2)	1 (12.5)	> 0.999	1 (11.1)	1 (12.5)	> 0.999
**Stoma formed**	38 (20.2)	30 (20.1)	8 (20.5)	> 0.999	6 (15.4)	8 (20.5)	0.555
**Theatre duration; min**	135.5 (29–560)	143 (29–560)	132 (49–327)	0.502	124 (29–301	132 (49–327)	0.682

*IBD* inflammatory bowel disease, *LARS* low anterior resection syndrome, *MEQ* morphine equivalents, *POD* postoperative day, *SB* small bowel

Postoperative outcomes are shown in Table 3. CCI was statistically higher in the SG than in the NSG (20.9 vs. 20.9, *p* = 0.047). No significant differences were found regarding CD grade, anastomotic leak, return to theatre, readmission or mortality rate. The SG had an increased rate of acute kidney injury (10.3 vs. 2.0%, *p* = 0.035) and trended towards more frequent ICU admissions (12.8 vs. 4.0%, *p* = 0.052). Overall sarcopenia was associated with a 1-day increase in the length of hospital stay (median 7 vs. 6 days, p = 0.028). The PSM cohort demonstrated no statistical difference in any complications and length of stay.

**Table 3:** Postoperative outcomes comparing non-sarcopenic and sarcopenic patients. Values are median (range) or number (proportion)

	**Total (*n* = 188)**	**Non-sarcopenic** **(*n* = 149)**	**Sarcopenic** **(*n* = 39)**	***P*-value**	**Matched non-sarcopenic** **(*n* = 39)**	**Matched sarcopenic** **(*n* = 39)**	***p*-value**
**Any complication**	128 (68.1)	96(64.4)	32 (82.1)	**0.036**	29 (74.4)	32 (82.1)	0.411
**CCI**	20.9 (0–71.6)	20.9 (0–70.1)	20.9 (0–71.6)	**0.047**	20.9 (0–61.7)	20.9 (0–71.6)	0.468
**CCI excluding no complications**	22.6 (9–71.6)	22.6 (9–70)	24.4 (9–71.6)	0.670	20.9 (9–62)	24.4 (9–71.6)	0.848
**CD grade**				0.098			0.703
No complication	60 (31.9)	53 (35.6)	7 (17.9)		10 (25.6)	7 (17.9)	
1–2	111 (59.0)	84 (56.4)	27 (69.2)		24 (61.5)	27 (69.2)	
3–5	17 (9.0)	12 (8.1)	5 (12.8)		5 (12.8)	5 (12.8)	
**ICU admission**	11 (5.9)	6 (4.0)	5 (12.8)	0.052	3 (7.7)	5 (12.8)	0.711
**Respiratory complication**	20 (10.6)	17 (11.4)	3 (7.7)	0.770	5 (12.8)	3 (7.7)	0.711
**Urinary complication**	16 (8.5)	13 (8.7)	3 (7.7)	> 0.999	4 (10.3)	3 (7.7)	> 0.999
**POI**	80 (42.6)	60 (40.3)	20 (51.3)	0.216	17 (43.6)	20 (51.3)	0.496
**Acute kidney injury**	7 (3.7)	3 (2.0)	4 (10.3)	**0.035**	1 (2.6)	4 (10.3)	0.358
**Cardiac complication**	10 (5.3)	8 (5.4)	2 (5.1)	> 0.999	3 (7.7)	2 (5.1)	> 0.999
**DVT/VTE**	4 (2.1)	4 (2.7)	0 (0.0)	0.582	0 (0.0)	0 (0.0)	
**Electrolyte disturbance**	23 (12.2)	20 (13.4)	3 (7.7)	0.420	4 (10.3)	3 (7.7)	> 0.999
**Anastomotic leak**	11 (5.9)	7 (4.7)	4 (10.3)	0.244	3 (7.7)	4 (10.3)	> 0.999
**Blood transfusion required**	9 (4.8)	5 (3.4)	4 (10.3)	0.091	0 (0.0)	4 (10.3)	0.115
**Return to theatre within 30-days**	13 (6.9)	10 (6.7)	3 (7.7)	0.735	4 (10.3)	3 (7.7)	> 0.999
**Readmission within 30-days**	27 (13.4)	20 (13.4)	7 (17.9)	0.473	7 (17.9)	7 (17.9)	> 0.999
**Mortality within 30-days**	0 (0.0)	0 (0.0)	0 (0.0)	–	0 (0.0)	0 (0.0)	–
**Length of stay; d**	6 (1–50)	6 (2–39)	7 (1–50)	**0.028**	6 (2–26)	7 (1–50)	0.153

*CCI* comprehensive complication index, *CD* Clavien-Dindo grade, *DVT* deep vein thrombosis, *ICU* intensive care unit, *POI* postoperative Ileus, *VTE* venous thromboembolism

Univariate and multivariate linear regression analyses was performed on all patients, determining factors predictive of CCI (Table 4). On univariate analysis, increased age, ASA ≥ 3, smoking history, sarcopenia and preoperative anaemia were associated with an increased CCI. However, age (*p* = 0.022), smoking history (*p* = 0.005) and preoperative anaemia (*p* = 0.008) remained predictive of CCI on multivariate linear regression analysis.

**Table 4:** Univariate and multivariate linear regression analysis for values predictive of CCI

	**β**	**95%CI**	**P-value**
**Univariate linear regression**			
Age ≥ 65	6.933	1.891–11.975	**0.007**
ASA ≥ 3	5.348	0.367–10.329	**0.035**
Active smoker	6.791	0.939–12.643	**0.023**
Sarcopenia	6.609	0.537–12.681	**0.033**
Preoperative anaemia (Hb < 110)	13.104	4.104–22.104	**0.005**
Variables not reaching significance—Gender, BMI ≥ 30, Dependence, CCF, COPD, HTN, DM, steroid use, previous abdominal surgery, MFI-5, preoperative hypoalbuminemia and total protein, surgical approach, conversion from laparoscopic, small bowel procedure			
**Multivariate linear regression**			
Age ≥ 65	6.023	0.881–11.164	**0.022**
ASA ≥ 3	2.669	−2.339 to 7.677	0.294
Active smoker	8.261	2.571–13.951	**0.005**
Sarcopenia	5.070	−0.749 to 10.889	0.087
Preoperative anaemia (Hb < 110)	12.078	3.249–20.907	**0.008**

*ASA* American Society of Anesthesiologists physical status, *BMI* body mass index, *CCF* congestive cardiac failure, *COPD* chronic obstructive pulmonary disease, *DM* diabetes mellitus, *HTN* hypertension requiring medication, *MFI* modified frailty index

## Discussion

Due to their catabolic metabolisms, compounded by systemic inflammation and malabsorption, colorectal cancer and severe inflammatory bowel disease has been the target of previous studies relating to sarcopenia [4, 6]. Shifting the focus to patients who have time to delay elective surgery, this study investigates the prevalence and morbidity of sarcopenia in patients with non-malignant colorectal disease. We diagnosed sarcopenia in one-fifth of our cohort, and despite showing an increase in complications when compared to the whole cohort, the PSM cohort showed no significant difference in complications and length of stay. This would suggest that if the focus of prehabilitation was shifted to non-malignant conditions, then it is unlikely that we will see significant improvements in overall complications.

When assessing our whole cohort, sarcopenic patients had a significantly higher rate of complications, including acute kidney injury (AKI) and postoperative ileus. Congestive cardiac failure and DM were more prevalent in the SG and increased their susceptibility to AKI. Increased frailty (defined as MFI-5 ≥ 2) was also more common in the SG and has previously been correlated with AKI following a surgical insult [18]. Our findings suggest that clinicians should be especially cautious with sarcopenic patients regarding fluid balance and the use of nephrotoxic agents, such as CT contrast. In addition to AKI, we found postoperative ileus (POI) complicated the postoperative course of 51.3% of sarcopenic patients. Defining POI remains challenging, and the strict definition used in our study may have contributed to our high rate of POI [19]. In the few relevant colorectal studies, a low incidence of < 6% POI in sarcopenic patients has been reported, with only a single study showing a rate of 25% [20, 21]. Our result is significantly higher and may represent selection bias, given that patients with complications are more likely to undergo radiological imaging investigations. This high rate of POI remained following PSM, suggesting the baseline characteristics of the studied cohort were more prone to developing POI. In an examination of colorectal cancer patients from our institution, we also confirmed there was an association between sarcopenia and the development of POI (OR 2.0, 95%CI 1.1–3.8) [22].

Given the waiting period prior to elective non-malignant colorectal surgeries, we hypothesise this time can be potentially utilised for preoperative optimisation targeting risk factors of poor outcomes. Despite being found as a risk factor on univariate analysis, sarcopenia was not demonstrated as a significant predictor of CCI on multivariate analysis. This was further confirmed by the PSM demonstrating no difference in postoperative complications, suggesting that the differences in our whole cohort were representative of the different baseline characteristics. However, we did reveal several other prognostic factors for increased CCI in non-malignant colorectal surgery that may be worth targeting. Risk factors include increased age (≥ 65 years old) and co-morbidities (ASA ≥ 3). Unfortunately, these are non-modifiable. Consistent with the literature, we also identified active smokers and preoperative anaemia as modifiable risk factors for poor outcomes after colorectal surgery [23, 24]. Active smokers had a strong correlation to increased CCI. Cessation of smoking forms an integral part of current prehabilitation and enhanced recovery protocols. This finding reaffirms that ongoing strategies to help patients stop smoking preoperatively will improve their short- and long-term outcomes [24]. In addition to smoking cessation, preoperative iron infusions for anaemic patients result in improved postoperative outcomes, decreased transfusion requirements and improved haemoglobin levels [23, 25]. In malignancy, anaemia may result from occult or overt gastrointestinal bleeding. Although the major proportion of patients in our non-malignant study cohort were not anaemic, when present, preoperative anaemia significantly impacted their postoperative outcomes. Consequently, we should be equally vigilant in screening for and preoperatively optimising anaemia in both our malignant and non-malignant colorectal surgery patients.

To date, the impact of prehabilitation in reducing morbidity following surgery is undefined. In a systematic review of 15 papers examining the effects of prehabilitation on complications following colorectal surgery, no reduction in clinically important complications was found (RR 1.02, 95% CI −0.79–1.31, *p* = 0.878) [7]. Furthermore, the review found no difference in the impact of exercise, nutrition and trimodal prehabilitation [7]. This review, and many of its included studies, grouped colorectal surgeries for malignant and non-malignant indications together. Consequently, it may grossly underestimate the efficacy of prehabilitation in patients with non-malignant disease, where the malabsorption and catabolic effects mediated by malignancy are not driving sarcopenia. Although a prehabilitation programme in non-malignant patients could potentially yield more promising improvements in skeletal muscle mass, our analysis only identified 39 patients who were sarcopenic out of the 188 patients identified over a 4-year period. Furthermore, the PSM cohort demonstrated no difference in complications. Thus, when considering the cost–benefit of implementing a prehabilitation programme targeting non-malignant colorectal surgery, based on our findings, we caution that this intervention may only benefit a small proportion of the overall colorectal surgical workflow and would have a limited impact on complications. Further work is required to develop optimisation and management strategies for sarcopenic patients undergoing colorectal surgery.

Our study has identified sarcopenia using the total psoas area index as a surrogate marker [13]. As demonstrated by Jones et al., the accuracy of this method has been shown to be comparable to computer calculated TPA. This method offers the benefit of being quick and easy for clinicians to use, allowing them to identify patients who likely have sarcopenia without requiring additional diagnostic radiological tools. However, it is important to note that this method does not consider fatty infiltration or myosteatosis, which have been shown to be a significant predictor of colorectal surgical outcomes [26, 27]. Other methods, such as examining bioelectrical impedance or measuring paraspinal and rectus muscles, in addition to total psoas area, can be used to establish a smooth muscle area as a marker of sarcopenia; however, these methods are not as timely as our chosen method [28].

This study has the inherent limitation of being a single-centred retrospective study with a low sample size. Additionally, the PSM only consisted of 78 patients. Nevertheless, given the paucity of data on the relationship between sarcopenia and benign colorectal disease, this study adds a significant contribution to the literature. Although identifying sarcopenia through radiological assessment of lean muscle mass is quick and easy, not all patients will have a preoperative CT scan. Furthermore, 42 patients were excluded due to no CT being performed, potentially underestimating the number of sarcopenic patients. Despite hand grip strength and the 6-min walk test being available to clinicians to diagnose sarcopenia, they are not routinely performed and remain a significant hurdle in identifying sarcopenia in benign colorectal disease. Additionally, we included a significant number of reversals of ileostomies, which have a differing incidence of complications compared to colonic surgeries. However, we deemed it important to include these cases as they constitute a substantial portion of non-malignant surgical procedures performed by a colorectal service. Small bowel procedures were also not predictive of CCI on univariate linear regression analysis. Prospective studies investigating sarcopenia in non-malignant elective surgery are required, given that this subset of patients has the potential to see the most improvement from a prehabilitation programme.

This study provides evidence that sarcopenia is present in a small proportion of non-malignant colorectal surgical cases but is not associated with increased morbidity in our analyses. Taking advantage of longer preoperative waiting periods, the focus should be placed on smoking cessation and treatment of preoperative anaemia. Future research is required to establish if prehabilitation programmes can improve surgical outcomes following non-malignant colorectal surgery.

## References

[1] Vergara-Fernandez O, Trejo-Avila M, Salgado-Nesme N (2020) Sarcopenia in patients with colorectal cancer: a comprehensive review. World J Clin Cases 8:1188–202. 10.12998/wjcc.v8.i7.1188 10.12998/wjcc.v8.i7.1188 PMC7176615 32337193

[2] Pin F, Couch ME, Bonetto A (2018). Preservation of muscle mass as a strategy to reduce the toxic effects of cancer chemotherapy on body composition. Curr Opin Support Palliat Care.

[3] Richards SJG, Senadeera SC, Frizelle FA (2020). Sarcopenia, as assessed by psoas cross-sectional area, is predictive of adverse postoperative outcomes in patients undergoing colorectal cancer surgery. Dis Colon Rectum.

[4] Trejo-Avila M, Bozada-Gutierrez K, Valenzuela-Salazar C, Herrera-Esquivel J, Moreno-Portillo M (2021). Sarcopenia predicts worse postoperative outcomes and decreased survival rates in patients with colorectal cancer: a systematic review and meta-analysis. Int J Colorectal Dis.

[5] Al-Khamis A, Warner C, Park J, Marecik S, Davis N, Mellgren A (2019). Modified frailty index predicts early outcomes after colorectal surgery: an ACS-NSQIP study. Colorectal Dis.

[6] Eros A, Soos A, Hegyi P, Szakacs Z, Benke M, Szucs A (2020). Sarcopenia as an independent predictor of the surgical outcomes of patients with inflammatory bowel disease: a meta-analysis. Surg Today.

[7] Zhang X, Wang S, Ji W, Wang H, Zhou K, Jin Z et al (2022) The effect of prehabilitation on the postoperative outcomes of patients undergoing colorectal surgery: a systematic review and meta-analysis. Front Oncol 12:958261. 10.3389/fonc.2022.958261 10.3389/fonc.2022.958261 PMC9372464 35965591

[8] Goates S, Du K, Arensberg MB, Gaillard T, Guralnik J, Pereira SL (2019) Economic impact of hospitalizations in US adults with sarcopenia. J Frailty Aging 8:93–9. 10.14283/jfa.2019.1010.14283/jfa.2019.1030997923

[9] Gani F, Buettner S, Margonis GA, Sasaki K, Wagner D, Kim Y (2016). Sarcopenia predicts costs among patients undergoing major abdominal operations. Surgery.

[10] Bedrikovetski S, Traeger L, Price TJ, Carruthers S, Selva-Nayagam S, Moore JW et al (2023) Can sarcopenia predict complete response after total neoadjuvant therapy in advanced rectal cancer? A multicentre observational cohort study. J Surg Oncol. 10.1002/jso.27251 10.1002/jso.27251 36971689

[11] Bedrikovetski S, Traeger L, Vather R, Sammour T, Moore JW (2022) Does sarcopenia predict local response rates after chemoradiotherapy for locally advanced rectal cancer? Dis Colon Rectum. 10.1097/dcr.0000000000002451 10.1097/DCR.0000000000002451 36538702

[12] von Elm E, Altman DG, Egger M, Pocock SJ, Gotzsche PC, Vandenbroucke JP (2007). The Strengthening the Reporting of Observational Studies in Epidemiology (STROBE) statement: guidelines for reporting observational studies. Lancet.

[13] Jones KI, Doleman B, Scott S, Lund JN, Williams JP (2015). Simple psoas cross-sectional area measurement is a quick and easy method to assess sarcopenia and predicts major surgical complications. Colorectal Dis.

[14] Fearon K, Strasser F, Anker SD, Bosaeus I, Bruera E, Fainsinger RL (2011). Definition and classification of cancer cachexia: an international consensus. Lancet Oncol.

[15] Clavien PA, Barkun J, de Oliveira ML, Vauthey JN, Dindo D, Schulick RD (2009). The Clavien-Dindo classification of surgical complications: five-year experience. Ann Surg.

[16] Slankamenac K, Graf R, Barkun J, Puhan MA, Clavien PA (2013). The comprehensive complication index: a novel continuous scale to measure surgical morbidity. Ann Surg.

[17] van Bree SH, Bemelman WA, Hollmann MW, Zwinderman AH, Matteoli G, El Temna S (2014). Identification of clinical outcome measures for recovery of gastrointestinal motility in postoperative ileus. Ann Surg.

[18] Ida S, Kaneko R, Imataka K, Murata K (2019) Association between sarcopenia and renal function in patients with diabetes: a systematic review and meta-analysis. J Diabetes Res. 10.1155/2019/1365189 10.1155/2019/1365189 PMC6885774 31828155

[19] Vather R, Trivedi S, Bissett I (2013). Defining postoperative ileus: results of a systematic review and global survey. J Gastrointest Surg.

[20] Nakanishi R, Oki E, Sasaki S, Hirose K, Jogo T, Edahiro K (2018). Sarcopenia is an independent predictor of complications after colorectal cancer surgery. Surg Today.

[21] Jochum SB, Kistner M, Wood EH, Hoscheit M, Nowak L, Poirier J (2019). Is sarcopenia a better predictor of complications than body mass index? Sarcopenia and surgical outcomes in patients with rectal cancer. Colorectal Dis.

[22] Traeger L, Bedrikovetski S, Nguyen TM, Kwan YX, Lewis M, Moore JW (2023). The impact of preoperative sarcopenia on postoperative ileus following colorectal cancer surgery. Tech Coloproctol.

[23] Leichtle SW, Mouawad NJ, Lampman R, Singal B, Cleary RK (2011). Does preoperative anemia adversely affect colon and rectal surgery outcomes?. J Am Coll Surg.

[24] Sharma A, Deeb AP, Iannuzzi JC, Rickles AS, Monson JRT, Fleming FJ (2013). Tobacco smoking and postoperative outcomes after colorectal surgery. Ann Surg.

[25] Froessler B, Palm P, Weber I, Hodyl NA, Singh R, Murphy EM (2018). The important role for intravenous iron in perioperative patient blood management in major abdominal surgery: a randomized controlled trial. Ann Surg.

[26] Herrod PJJ, Boyd-Carson H, Doleman B, Trotter J, Schlichtemeier S, Sathanapally G (2019). Quick and simple; psoas density measurement is an independent predictor of anastomotic leak and other complications after colorectal resection. Tech coloproctol.

[27] Tankel J, Yellinek S, Vainberg E, David Y, Greenman D, Kinross J (2020). Sarcopenia defined by muscle quality rather than quantity predicts complications following laparoscopic right hemicolectomy. Int J Colorectal Dis.

[28] Sasaki M, Fukuoka T, Shibutani M, Sugimoto A, Maeda K, Ohira M (2022). Usefulness of the skeletal muscle index in postoperative ileus of colorectal cancer patients: a retrospective cohort study. BMC Surg.

